# Anaplastic large cell lymphoma as a posttransplant lymphoproliferative disorder in a renal transplant patient: A case report

**DOI:** 10.1002/jha2.13

**Published:** 2020-07-10

**Authors:** Manar Alabdulbaqi, Melissa Toupin, Philip Berardi, Arleigh McCurdy

**Affiliations:** ^1^ Department of Hematology The Ottawa Hospital Ottawa Ontario Canada; ^2^ Department of Hematology Ottawa Hospital General Campus Ottawa Ontario Canada; ^3^ Department of Anatomic Pathology/Hematopathology Ottawa Hospital General Campus Ottawa Ontario Canada; ^4^ Department of Hematology University of Ottawa Ottawa Ontario Canada

## Abstract

We report a case of a 45‐year‐old female who developed an ALK‐positive anaplastic large cell lymphoma (ALCL) 9 years after renal transplant. The patient underwent a cadaveric renal transplant for diabetic nephropathy, and presented 9 years later with fever and multiorgan dysfunction. The initial CT scans showed multiple enlarged supra‐ and infradiaphrgamatic lymph nodes. A CT‐guided core needle biopsy of a retroperitoneal lymph node revealed ALK positive ALCL. She received six cycles of cyclophosphamide, adriamycin, vincristine, etoposide, and prednisone, and has been in remission for over 3 years. Monomorphic T‐cell posttransplant lymphoproliferative disorder (PTLD) is an established but rare entity of PTLD and generally carries poor prognosis. This is a case report of a late PTLD with pathology reporting an aggressive T‐cell lymphoma that has been successfully treated with multiagent chemotherapy.

## 1 BACKGROUND

Post‐transplant lymphoproliferative disorder (PTLD) is a potentially fatal malignancy arising after solid organ or hematopoietic stem cell transplant. Iatrogenically impaired immune surveillance and Epstein‐Barr virus primary infection/reactivation are key factors in the pathogenesis.^1^ The incidence of PTLD depends on the transplanted organ. Recipients of renal transplantation have the lowest incidence (0.8‐2.5%), followed by pancreatic transplant (0.5‐5%), liver transplant (1‐5.5%), heart transplant (2‐8%), lung transplant (3‐10%), and multiorgan and intestinal (about 20%). They usually occur during the first year. A second peak occurs 5 to 15 years after transplant and cases tend to be EBV negative.^2^

Based on World Health Organization 2016 classification, there are 4 categories of PTLD: Non‐destructive PTLD (previously called early PTLD), which includes Plasmacytic Hyperplasia, infectious mononucleosis, and florid follicular hyperplasia. The second category is Polymorphic PTLD, which remains pathologically challenging to diagnose. The third category is Monomorphic PTLDs, which are classified according to the lymphoma they resemble. The latter includes both B and T cell neoplasia. A fourth category is assigned to classic Hodgkin lymphoma.^3^

PTLD carries a poor prognosis. Unfortunately, there is paucity of prospective data regarding the best approach to treatment. In the setting, PTLD of T cell type options include reduction of immune suppression and multiagent chemotherapy.

## 2 CASE REPORT

This is a 45‐year‐old female with type 1 diabetes complicated by retinopathy and nephropathy. She received a cadaveric renal transplant for advanced diabetic nephropathy and was started on post‐transplant immunosuppression with tacroliums, mycophenolate mofetil and prednisone. She is known to have coronary artery disease, hypertension, gastroesophageal reflux and depression/anxiety.

Nine years following transplant, she had a prolonged hospital admission for fever of unknown origin complicated by respiratory failure requiring intubation and vasopressor support. She eventually developed liver and renal failure. Prior to that, she had stable graft function and was kept on a low dose prednisone (5 mg daily), azathioprine (100 mg daily), and extended release tacrolimus (1.5‐2 g daily). Physical examination was negative for palpable lymphadenopathy and organomegaly. Basic investigations revealed profound anemia of 69 g/L, reference range RR of (115‐155 g/L), White blood cells of 2 × 10^9^/mL RR (3.5 × 10^9^), and platelets of 44 × 10^9^ /mL RR (130–380 × 10^9^) in the context of sepsis. Lactate dehydrogenase was 395 U/L, RR (100–205). Computed tomography CT scans showed enlarged paratracheal lymphadenopathy measuring, small volume retroperitoneal lymphadenopathy including: left para‐aortic lymph node, right common iliac node, right external iliac node. The spleen measured 12.8 × 8.7 cm. CT guided biopsy of the right iliac lymph node was obtained.

**Figure:**
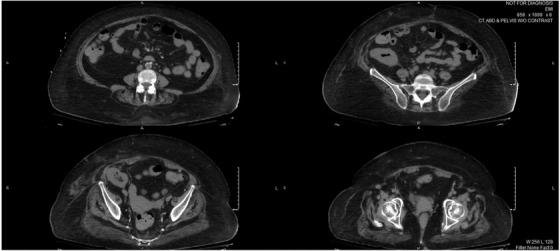
CT images with no contrast showing enlarged para‐aortic lymph nodes.

The pathology showed an infiltrate of large cells with eccentric, horseshoe‐ or kidney‐shaped nuclei often with an eosinophilic region near the nucleus. The “Hallmark” cells were present in a relatively high abundance and showed significant variability in their overall morphology. They contained abundant cytoplasm and some cells showed a pattern of peripheral clearing. Reed‐Sternberg like cells were not a prominent feature. The nuclear chromatin was dispersed with prominent nucleoli. The tumor cells appeared to be infiltrating the lymph node sinuses. There were numerous reactive histiocytes and small lymphocytes in the background.

The large cell infiltrate was strongly positive for CD4, CD30, ALK, CD25, CD43, CD45 (LCA), MUM1, EMA and Vimentin. Staining for TIA‐1 was positive in a small subset of the large cells of interest. Staining for BCL6 was positive and staining for BCL2 shows weak cytoplasmic expression in the large neoplastic cells of interest. The infiltrate was negative for CD3, CD5, CD8, CD15, CD20, PAX5, CD79, CD68, CK‐PAN, CD10, CD23, cyclin D1 and S100. Staining for c‐MYC was positive in approximately 80% of large cells; staining for p53 was variable and estimated at 10%. The Ki67 labeling was >80%. Staining for EBV by EBV was negative. The diagnosis was Post‐transplant lymphoproliferative disorder with features of ALK‐positive ALCL.

Bone marrow aspirate and biopsy were obtained and showed increased histiocytes with hemophagocytosis suggestive of Hemophagocytic Lymphohistyocytosis (HLH) but no lymphoma cell infiltrate. Trephine biopsy was of compromised quality and was not reportable.

Other investigations revealed a fibrinogen of >16,000 µg/L, RR(1.9–4.5 µg/L), and Triglycerides of 4.3 mmol/L, RR (≤1.7 mmol/L). She therefore has fulfilled at least 4 of the HLH criteria and was subsequently labeled secondary HLH in the settings of PTLD.^4^

Patient's immunosuppression was reduced to Prednisone 5 mg orally daily and tacrolimus 1.5 to 2 mg orally daily. She did maintain a good renal function.

**Figure:**
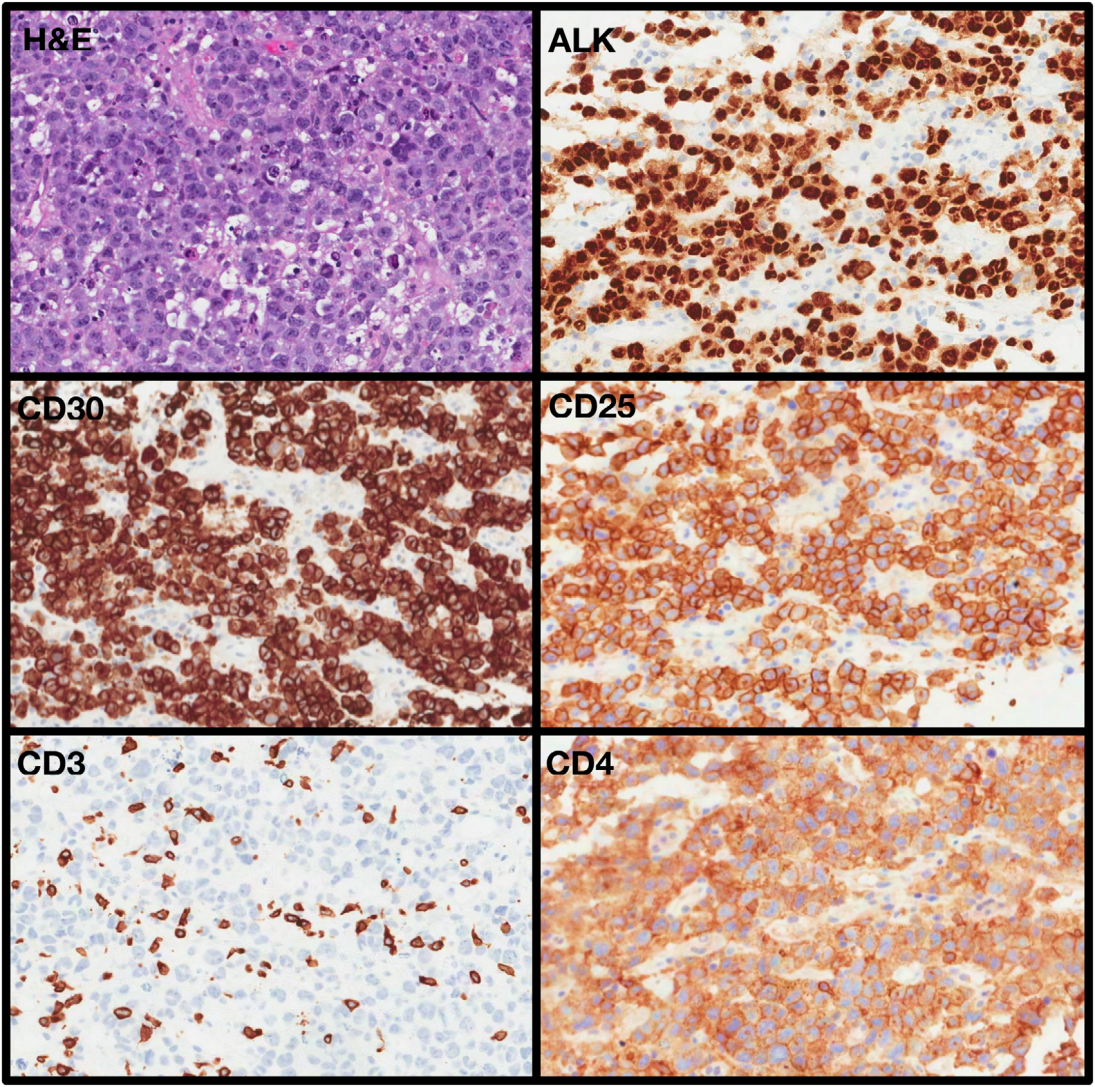
H&E slide and immunohistochemical stains of the lymph node

The patient received chemotherapy CHOEP (cyclophosphamide, doxorubicin, vincristine, Etoposide and prednisone) × 6 cycles with granulocyte‐colony stimulating factor started 24 hours after last chemotherapy. Chemotherapy was well tolerated. Post chemotherapy positron emission tomography–CT was planned but was not done due to poorly controlled blood sugar. A repeat CT scan abdomen and chest with contrast showed regression of abdominal lymphadenopathy. Her LDH has normalized to 184 by the end of her treatment and fever has subsided. Patient has been in clinical remission ever since.

## 3 DISCUSSION

Posttransplant lymphoproliferative disorder is a well‐recognized complication of solid organ and hematopoietic stem cell transplant. Early onset PTLD happens during the first year posttransplant and is typically seen in EBV positive recipients, whereas late onset occurs 5–15 years posttransplant and is associated with EBV negative recipients. PTLD has a universally poor prognosis and the current approach in practice is mainly based on expert opinion due to a lack of robust evidence.

EBV positivity is not a prerequisite for the diagnosis of PTLD, and the number of EBV negative cases has increased over time from 10% (1990‐1995) to 48% (2008‐2013).^1^ The mechanisms that cause EBV‐negative PTLD are not well understood. This disorder often has a later onset, monomorphic histology, and a more aggressive course than EBV‐positive PTLD, suggesting that it may have a different pathophysiology.

There are few cases reported of ALCL‐PTLD. In a relatively recent retrospective cohort study of 227 adult renal transplant recipients followed in two transplant centers in Hong Kong over two decades, 28 patients developed PTLD with only 1 patient diagnosed with T Cell Lymphoma of unspecified entity. The index patient failed CHOP (cyclophosphamide, Doxorubicin, vincristine and prednisone) and died 14 months after the diagnosis due to sepsis.^5^ Data collected from the French Registry enrolling all adult renal transplant recipients who developed PTLD between January 1998, and December 2007 reported 500 cases of PTLD with T‐cell lymphomas accounting for 6% of the total number (n = 29).^6^ A case series was published in 2007 by Magro et al of 2 patients with T‐cell‐associated cutaneous ALCL in the setting of liver transplant. The authors concluded that these cases did not appear to be directly attributable to EBV infection. Iatrogenic immune dysregulation may result in excessive T‐cell proliferation to various antigenic stimuli, hence resembling other drug‐associated cell lymphoproliferative conditions.^7^

A case report was published in 2011 on a case of ALCL‐PTLD presenting in the gall bladder. The patient died due to acute cholecystitis prior to receiving any lymphoma specific treatment.^8^ In our literature search, there was only one published case of a successfully treated ALCL in the setting of PTLD of a 13 year old boy treated with reducing his immune suppression and chemotherapy: ACVBP that includes Doxorobucin, Cyclophosphamide, Vincristine, bleomycin, and Prednisone for 6 cycles with preservation of his graft function.^9^ Of interest, Willenbacher et al published a case of a late ALCL in the setting of lung transplant. The patient was too frail for multi‐agent chemotherapy and the treating group opted for an off label use of Brentuximab Vedotin, which was well tolerated. Interim evaluation showed complete metabolic remission and patient completed 3 cycles without significant complications at the time the case report was published.^10^

First line therapy is usually reduction of immunosuppression, which potentially carries the risk of compromising graft function and eventually graft rejection. Chemotherapy, typically CHOP with or without Rituximab, is usually reserved for cases that fail frontline immunosuppression reduction and Rituximab monotherapy, or, for those who present with fulminant PTLD including central nervous system lymphoma.

More recently, ECHELON2 trial published in 2018 has demonstrated that Brentuximab in addition to cyclophosphamide, Doxorubicin and prednisone (A+CHP) had superior progression free survival (PFS) and overall survival (OS) compared to CHOP protocol. Eligible patients were those who had previously untreated CD30 positive T cell lymphoma, 75% of which were ALCL. The study did not specify the background of the patient population. However, we may extrapolate that it is likely effective in the PTLD cohort. This is limited by the paucity of data in the latter and potentially the lack of funding at many treating institutions.^11^

To our knowledge, there are no prospective trials addressing the best approach to PTLD with T cell PTLD‐ due to the relatively low incidence and the heterogeneity of the patient population. This case report is one of few reports of a patient with late onset ALCL‐PTLD successfully treated with multiagent chemotherapy.

## CONFLICT OF INTEREST

The authors declare that there is no conflict of interest.
